# Influence of Sublethal Carbon Dioxide on Biological Characteristics and Life Table Parameters of *Cynaeus angustus* (LeConte) (Coleoptera: Tenebrionidae)

**DOI:** 10.3390/insects17060576

**Published:** 2026-05-31

**Authors:** Ruotong Shen, Dianxuan Wang, Chen Wang, Xi Zhu, Huanyi Sun, Qiaozhen Zhang

**Affiliations:** Grain Storage and Logistics National Engineering Research Center, National Grain Industry (Storage Insect Pest Control) Technology Innovation Center, Henan University of Technology, Zhengzhou 450001, China; 18638707670@163.com (R.S.); 18638764239@163.com (C.W.); zhu_xi537@163.com (X.Z.); shyshallop@126.com (H.S.); zhenqiao1008@163.com (Q.Z.)

**Keywords:** *Cynaeus angustus*, sublethal carbon dioxide, survival rate, development duration, life table parameters

## Abstract

Understanding insect survival and developmental characteristics in sublethal carbon dioxide (0.25–4%), which could exist in stored grain, is beneficial to scientific pest management. The survival, developmental duration, oviposition, and life table parameters of *Cynaeus angustus* (LeConte) were measured with carbon dioxide at 0.25%, 0.5%, 1%, 2%, and 4% in air, respectively, and 0.04% as a control. The developmental duration at 4% of carbon dioxide was 87 days versus 90 days in control at 23 °C, 79 days versus 85 days in control at 28 °C. The generation survival rate varied from 77% to 69% at 23 °C, and from 81% to 74% at 28 °C in the concentrations. The number of eggs laid per female reduced from 28 in the control to 19 in 4% of carbon dioxide. The sex ratio decreased from 1.19 and 1.21 to below 1.0 at 4% of carbon dioxide at the tested temperatures. Life table parameters (*T*, *R*_0_, *r*, *λ*) were significantly decreased at and above 0.5% carbon dioxide at 23 °C; *r* and *λ* were significantly decreased at 2% carbon dioxide at 28 °C. Carbon dioxide above 0.25% or 0.5% significantly affected the survival and development, which may affect fumigation or controlled atmosphere with this sublethal carbon dioxide.

## Simple Summary

Carbon dioxide concentration of 0.25–4% has been measured in stored grain loaded initially or infested with pests. Understanding insect survival and developmental biology under sublethal concentrations of carbon dioxide that can be monitored in grain storage may give some biological reference in pest management, especially for fumigation or controlled atmosphere, in which carbon dioxide could synergize the effect of insect killing. *Cynaeus angustus* has been developing distribution around the world. The measurement of some biological parameters was carried out when *C. angustus* was exposed to 0.25, 0.5, 1, 2, and 4% of carbon dioxide at 23 °C and 28 °C and compared with the control treatment (ambient air). The concentration of carbon dioxide at 0.25–4% significantly affected the survival and development of *C. angustus* both at 23 °C and 28 °C. The generation duration, survival rate, oviposition, and life table parameters (*R*_0_, *r*, *λ*, and *T*) changed significantly when carbon dioxide exceeded 0.25% or 0.5%. The results indicate that variation in the survival ability, developmental duration and other biological parameters of *C. angustus* should broaden knowledge and inform management practices, such as fumigation and controlled atmosphere, where exposure time can be affected by survival and developmental parameters of *C. angustus*.

## 1. Introduction

The survival and developmental biological parameters of stored product pests are the basis of scientific pest management [1,2]. Insect developmental and survival parameters especially affect the exposure time of fumigation or controlled atmosphere [3]. Life table parameters of insects may also reflect responses to abiotic factors [4]. Many studies have reported on the survival, growth, development, fecundity, and population dynamics of stored product pests and examined the effects of factors such as temperature, relative humidity, and food type (e.g., [2,5,6,7,8,9]). Insect pests in stored products can cause significant damage and losses [10,11,12], highlighting the importance of effective pest management.

The carbon dioxide in stored grain can be monitored [13,14,15], which could be utilized for insect and micro-organism monitoring, even to synergize the effect of fumigation or controlled atmosphere [16,17]. Grain, microorganisms, and insect pests can produce obvious carbon dioxide in grain mass in many cases [18,19], especially in large scale of the bulk and sealed warehouses [20]. The atmospheric carbon dioxide concentration is approximately 0.04% in ambient air usually. Carbon dioxide concentration had increased to 4.0% at 20–30 °C after 180 days of storage in wheat with 12–13% grain moisture content (m.c.) [21]. And a significant accumulation of carbon dioxide was also detected in wheat bulk with 18% m.c. [22]. Carbon dioxide reached 0.07% in soybean storage with 12.7% m.c. within 30 days [23], increased to 0.4% after 128 days, and further rose to 4.2% after 206 days in silo bags [24]. Carbon dioxide concentrations in stored corn reached 0.22% at 16% m.c. and 0.75% at 18% m.c. within 60 days [20]. The respiration rate of maize increased exponentially when the grain moisture content exceeded 16.5% at 25 °C, indicating that stored grain can produce more carbon dioxide at higher moisture contents [25].

The survival and development of insects may be influenced by carbon dioxide. For example, a significant reduction in oviposition ratios and an extension of immature developmental periods were observed in *Tribolium castaneum* (Herbst), *Cryptolestes pusillus* (Schnherr), and *Cryptolestes ferrugineus* (Stephens) following exposure to carbon dioxide concentrations of 7.5% or 8.6% for 1 to 3 weeks [26]. Carbon dioxide may also act synergistically with low oxygen conditions, as carbon dioxide at 2–8% combined with oxygen at 2–4% (in contrast to 21% in ambient air) significantly increased mortality and reduced pupation and adult emergence of *Plodia interpunctella* (Hübner) [27]. Understanding the survival and developmental characteristics of insects at sublethal carbon dioxide should help to know the survival ability of insects in the special grain bulk with carbon dioxide metabolized by biological factors and to support the synergistic use of fumigation and controlled atmosphere [28,29].

The larger black flour beetle, *Cynaeus angustus* (LeConte) (Coleoptera: Tenebrionidae), is a pest species whose distribution is growing, thus increasing its economic importance in the grain storage industry around the world [30,31]. In the past four decades, *C. angustus* has been recorded throughout the continental United States [32,33], recorded in Sweden and Finland [30], France [31], Ukraine and Russia [34], China [35], South Korea [36], Germany [37], Poland [38], the Czech Republic [39], and Romania [40]. This insect had spread throughout many countries and caused damage to stored grains, flour and related products, especially preferring maize [33].

The influence of temperature on development, movement, population dynamics, fecundity, survival, generation duration, population size, and geographic range in ambient air has been reported in many studies [33,41,42]. Low carbon dioxide (such as 0.25–4%) could be accumulated due to grain respiration and pest activity, and are increasingly used as a monitoring indicator in more and more modern grain storage facilities. The influence of sublethal carbon dioxide levels on survival, development, and population performance of *C. angustus* is still lacking in reports. The survival, developmental duration, oviposition, and life table parameters of *C. angustus* at 0.25, 0.5, 1, 2, and 4% of carbon dioxide at 23 °C and 28 °C were measured. This study provides biological characteristics of how carbon dioxide and temperature affect *C. angustus*, which can provide information for grain monitoring and improving fumigation or controlled atmosphere practices.

## 2. Materials and Methods

### 2.1. Measurement Device

**Figure 1:**
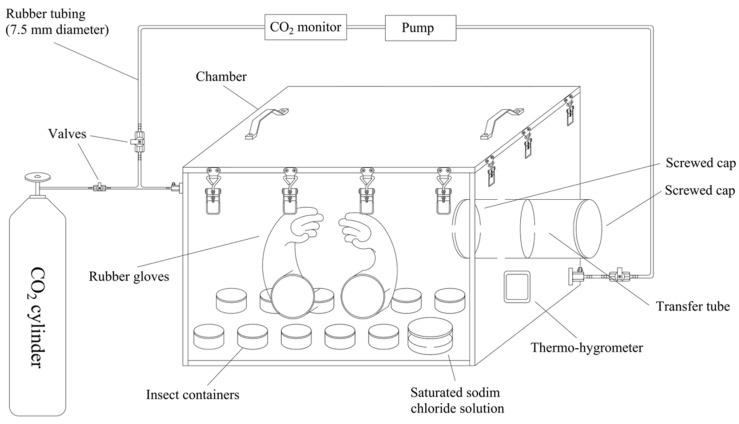
Diagram of the measurement device.

The chamber (60 cm × 35 cm × 40 cm) was made of 2 mm thick transparent acrylic plate (Figure 1) that was used to provide carbon dioxide concentrations. The half-life time from 500 Pa to 250 Pa was 180 s in airtightness tests. Two rubber gloves with 150 mm-diameter connecting holes were installed on the wall of the chamber, and were used to operate insect samples and arrange other manipulations. A transfer tube was installed through another wall of the chamber (100 mm in inner diameter × 140 mm in length). Both ends of the tube could be sealed or opened with screwed caps. Insect samples could be placed into, removed from, or raised for observation by hand through the gloves and the transfer tube. The gas in the chamber was recirculated and monitored daily using a carbon dioxide monitor equipped with a pump (MS400-XH-2; detection range: 0–20% carbon dioxide; precision: ±0.01%; Shenzhen Yiyuntian Electronics Co., Ltd., Shenzhen, China).

### 2.2. Insects

The population of *C. angustus* here was collected from a grain depot in Zhengzhou, China, reared with cracked maize sieved through a 4.0 mm mesh and yeast powder (95:5, *w*/*w*). Eggs were obtained from 100 unsexed 7-day-old adults, and the diet was provided in plastic containers (8 cm in diameter × 1.5 cm in height), where black craft paper was used to collect the sticky eggs. Each egg, stuck on a small paper piece that was cut by scissors, was placed individually into plastic containers (21.5 mm in diameter × 10 mm in height).

The insect containers placed on the bottom of the chamber were observed through the transparent acrylic using a handheld LED magnifier (40× magnification; RSJ.3008C; Zhongbang Optical Instrument Co., Ltd., Yiwu, China). Fifty one-day-old eggs were used for observation in the chamber. Each egg was individually put in a plastic container (21.5 mm in diameter × 10 mm in height), which was placed in the chamber. Three parallel chambers were used for each measurement. The 75 ± 5% r.h. in the chamber was maintained using a saturated sodium chloride solution in a 250 mL beaker. The chambers were placed in rooms where temperatures were controlled by air conditioners and continuously monitored at 23 ± 1 °C and 28 ± 1 °C, respectively.

### 2.3. Measurement of Survival and Development

Carbon dioxide of 0.25%, 0.5%, 1%, 2%, and 4% in air was controlled by releasing liquid carbon dioxide (99.99%) from a cylinder. The concentration was measured and adjusted daily in the process. Ambient air (carbon dioxide at 0.04%) was used as a control. Egg hatching was recorded daily. The diet (2 g in weight) was added to newly hatched larvae using a fine brush through the rubber glove operation. Each insect was observed through the top transparent plate of the chamber with a handheld LED magnifier. A small fine brush placed inside the chamber was used to gently separate the diet and the insects. During observation, the insect containers were moved up and down by hand through the rubber gloves to a clear view of each individual. Larval molting, survival, and developmental duration, pupation duration, pupal survival, eclosion duration, and adult survival were recorded daily. The generation duration was determined from egg to adult emergence. The non-moving larva or adult was confirmed to be dead when touched on the abdomen with a fine brush.

### 2.4. Oviposition and Sex Ratio

Adults used for oviposition assessment were obtained from the corresponding carbon dioxide treatments. For each treatment, ten pairs of newly emerged adults were selected and individually placed in separate plastic containers with a 2 g diet. The containers were kept in the same concentration and temperature as before. Eggs laid by each female were counted daily until oviposition ceased. The gender was distinguished by the caudal spine of pupae, in which the caudal spine of males is longer and sharper. The sex ratio was calculated with the number of female to male individuals.

### 2.5. Data Analysis

The developmental duration, survival rate, oviposition, sex ratio, and life table parameters of *C. angustus* were analyzed using the age-stage, two-sex life table model with the computer program TWOSEX-MSChart [42,43] (http://140.120.197.173/Ecology/Download/Twosex-MSChart.rar, accessed on 18 May 2026). The means and standard errors of these parameters were estimated using the bootstrap method with 100,000 resamplings [44], and the paired-bootstrap test was used to assess the differences among treatments. Differences between temperatures within the same carbon dioxide concentration were tested using an independent samples *t*-test for sex ratio.

The survival rate (%) was calculated as follows:

(1) $$Survival\;rate\;(\%)=\frac{Number\;of\;surviving\;individuals}{50}\times 100\%$$

Generation survival rate was calculated as follows:

(2) $$Generation\;survival\;rate\;(\%)=\frac{Number\;of\;emerged\;adults}{50}\times 100\%$$

The following life table parameters were calculated as [45,46]:

(3) $$\mathrm{Net}\;\mathrm{reproductive}\;\mathrm{rate}\;(R_{0}):\;R_{0}=\Sigma \;l_{x}\;m_{x}$$

(4) $$\mathrm{Intrinsic}\;\mathrm{rate}\;\mathrm{of}\;\mathrm{increase}\;(r):\;r=\Sigma e^{-r(x+1)}\cdot lₓ\;mₓ=1$$

(5) Finite rate of increase (λ): e^(r)

(6) $$\mathrm{Mean}\;\mathrm{generation}\;\mathrm{time}\;(T):\;T=ln(R_{0})\;/\;r$$

*x*: developmental age; *l_x_*: survival rate of *C. angustus* during age *x*; *m_x_*: number of eggs laid per female during age *x*. Data obtained from three independent chambers for each carbon dioxide concentration were treated as biological replicates for statistical analysis.

## 3. Result

### 3.1. Developmental Duration and Adult Longevity

At 23 °C, the egg duration was 4.12 ± 0.07 days in the control and ranged from 4.06 ± 0.48 to 3.51 ± 0.06 days in the tested carbon dioxide concentration. There were no significant differences between the control and the treatments (*p* > 0.05, Table 1). The developmental duration of larval instars (L1–L12) was significantly affected only by carbon dioxide at 4% compared with the control and other concentrations (*p* < 0.05), except for L2, which was affected by 2% and 4% of the concentration. The duration of the larval stage was significantly shortened by 8.23–10.29 days as carbon dioxide increased from 2% to 4%. This shortening indicates a cumulative effect of carbon dioxide on larval development across all larval instars (L1–L12). The pupae and the generation duration, and adult longevity were significantly decreased in carbon dioxide above 2% (*p* < 0.05). And no significant difference in these parameters was measured between 2% and 4% of the concentration (*p* > 0.05, Table 1).

At 28 °C, the egg duration was 3.15 ± 0.74 days in the control and ranged from 2.46 ± 0.65 to 2.98 ± 0.21 days in the treatments, in which the duration was shortened by about 0.97–1.14 days compared with that at 23 °C (Table 2). The difference in carbon dioxide concentration had no significant impact on the egg stage (*p* > 0.05). The duration of L2 to L12 varied significantly in carbon dioxide at 2% and 4% (*p* < 0.05). The duration of larval stages changed more in response to carbon dioxide at 28 °C than at 23 °C, except for L1. The duration of the total larval stage and the generation duration was significantly shortened in carbon dioxide at 1% (*p* < 0.05), whereas there were no significant differences in pupal development duration (*p* > 0.05). The longevity of male and female adults did not differ significantly in the control or in carbon dioxide at 0.25% to 1% (*p* > 0.05) but had a significant reduction in carbon dioxide above 2% (*p* < 0.05, Table 2). The pupal duration at 28 °C was shortened by approximately 1.35–1.82 days, and the generation duration was significantly reduced by about 6.14–8.82 days, compared with that at 23 °C. At 28 °C, the longevity had increased approximately 10.71–12.77 days for females and increased approximately 10.57–12.80 days for male adults, compared with that at 23 °C (Table 2).

### 3.2. Survival Rates

At 23 °C, the egg survival rate was 94.72 ± 1.02 in the control and 94.10 ± 0.96 to 91.12 ± 0.94 in the carbon dioxide concentration of 0.25% to 4%, and there was a significant decline in carbon dioxide at 2% (*p* < 0.05, Table 3). The survival rate of L1 to L12 was significantly affected by carbon dioxide at 0.25%, except for L1–L3, which was affected at a concentration of 0.5%. The survival rate of the total larval stages was decreased significantly when carbon dioxide was above 0.25%, while the survival rate of pupae and the generation duration were decreased significantly when carbon dioxide was 0.5% (*p* < 0.05, Table 3).

At 28 °C, the significant differences in survival rates were measured in the egg stage in carbon dioxide at 2% (*p* < 0.05). The significant differences were detected in the survival of L1–L2, L7–L9, L12, and the pupal stage in concentration of 0.5%, and were observed in the survival of L3–L6, L10–L11, and the total larval stage at 0.25% of the concentration (*p* < 0.05, Table 4).

The survival rates in developmental stages of *C. angustus* were generally higher at 28 °C than at 23 °C. Contrastively, the survival rates of the different life stages of *C. angustus* were affected differently under carbon dioxide ranging from 0.5% to 4% at 28 °C, with greater reductions detected at higher concentrations.

### 3.3. Sex Ratio

The sex ratio of *C. angustus* changed significantly from 1.19 ± 0.03 under the control to 0.87 ± 0.02 at 4% of carbon dioxide at 23 °C (Table 5). The sex ratio under 0.25% and 0.5% of carbon dioxide was significantly lower than that in the control (*p <* 0.05), and decreased further significantly under 1%, 2%, and 4% of carbon dioxide (*p <* 0.05, Table 5). There were no significant differences in sex ratio among the carbon dioxide at 0.25%, 0.5%, and 1%, compared with that in the control at 28 °C (*p* > 0.05, Table 5). The sex ratio decreased significantly under 2% carbon dioxide and was the lowest of sex ratios under 4% of carbon dioxide (*p <* 0.05, Table 5), which means that carbon dioxide at the tested concentrations can decrease the female ratio of *C. angustus* both at 23 °C and 28 °C (Table 5). The sex ratio at 28 °C was 0.09 higher than that at 23 °C at carbon dioxide of 2% and was 0.06 higher at carbon dioxide of 4% than that at 23 °C (*p <* 0.05, Table 5).

### 3.4. The Oviposition and Life Table Parameters

At 23 °C, the pre-oviposition duration was significantly prolonged at 1% of carbon dioxide compared with that in the control (*p* < 0.05, Table 6). The oviposition duration was significantly prolonged at 2% and 4% of the concentration (*p* < 0.05, Table 6). The number of eggs laid per female declined significantly at 0.25% of carbon dioxide and decreased further with increasing the concentration (*p* < 0.05, Table 6). The significant decrease was measured in carbon dioxide at 0.25% and more for *R*_0_, at and above 0.5% of the concentration for *r*, *λ* and *T* (*p* < 0.05, Table 6).

At 28 °C, the pre-oviposition duration was significantly prolonged at 2% of carbon dioxide compared with that in the control (*p* < 0.05, Table 7), and the oviposition duration was significantly prolonged at 2% and 4% of the concentration (*p* < 0.05, Table 7). The number of eggs laid per female declined significantly at 0.25% of carbon dioxide (*p* < 0.05, Table 7). Significant decline was observed at 0.25% of carbon dioxide for *R*_0_, at 2% of the concentration for *r* and *λ*, and at 0.5% of the gas for *T* (*p* < 0.05, Table 7).

Compared with 23 °C, the pre-oviposition duration at 28 °C was shortened by 1.04–1.10 days, the oviposition duration was prolonged by 1.78–2.52 days, and the number of eggs laid per female increased by 1.24–1.50. The values of *R*_0_, *r*, and *λ* at 23 °C were generally lower than those at 28 °C in all treatments (Table 6 and Table 7). The *R_0_* value at 28 °C was 0.69–1.18 higher than that at 23 °C, while *T* at 28 °C was shortened by 7.30–9.20 days compared with that at 23 °C under the control and all tested concentrations (Table 6 and Table 7).

## 4. Discussion

The developmental duration of the generation of *C. angustus* was 92 days at 23 °C and 86 days at 28 °C in the control, respectively. The larval development duration was 80 days at 23 °C and 69 days at 28° in the control (Table 1 and Table 2). When carbon dioxide increased from 0.25% to 4%, the generation developmental duration decreased from 90 to 87 days at 23 °C, and from 83 to 79 days at 28 °C, respectively. The total generation developmental duration of *C. angustus* in the tested carbon dioxide concentrations mainly depended on the larval development at the two temperatures. The previous studies reported that larvae are the most active feeding stage and often account for the largest proportion of the immature period, thereby strongly influencing the total generation developmental duration [47,48,49].

The whole generation survival rate of *C. angustus* was 78% at 23 °C and 81% at 28 °C in the control, varied from 76% to 69% at 23 °C and from 80% to 74% at 28 °C, correspondingly, with carbon dioxide increasing from 0.25% to 4% (Table 3 and Table 4). The whole generation survival rate was mostly affected by the larval survival rate. Furthermore, changes in larval survival and larval developmental time can directly alter generation survival and ultimately influence population growth [50]. Which is similar to that of carbon dioxide at 18% significantly increased the mortality of L1 and L4 of *Callosobruchus maculatus* (Fabricius) [51]. Otherwise, the survivors suffering the sublethal concentration of carbon dioxide exhibited faster development compared to those in ambient air. The effects of carbon dioxide at 0.25–4% on *C. angustus* were similar to those detected under low oxygen conditions combined with elevated carbon dioxide at comparable levels [26].

Insect development and oviposition could be stressed by adverse factors such as low temperature and humidity, modified atmosphere conditions (e.g., elevated carbon dioxide or low oxygen), and exposure to insecticides or fumigants [52,53,54]. The oviposition of *C. angustus* females was significantly reduced in carbon dioxide at 0.25–4%, compared with that in the control. Meanwhile, the proportion of females declined under sublethal carbon dioxide, which is consistent with a previous report that insects can survive but exhibit altered biological characteristics under sublethal carbon dioxide at 2%, 5%, and 10% [55]. At a carbon dioxide concentration of 18% in a controlled atmosphere, cowpea weevil could finish their development and reproduction, but their mortality increased and oviposition decreased significantly [51]. The reason for the female ratio reduction of *C. angustus* in the tested carbon dioxide may be related to energy consumption or detoxification between females and males [56]. Females consumed more energy and were more susceptible to sublethal carbon dioxide than males because respiration may impose higher energetic demands. This is similar to the findings in *Dendroctonus armandi*, where a female-biased sex ratio was associated with energy storage and consumption [57].

Life table parameters are important indicators for evaluating insect adaptability to environmental conditions [58,59]. The innate rate of increase (*r*) and the finite rate of increase (*λ*) are parameters to measure the future trends of population increasing or decreasing [60]. Compared with *Tribolium castaneum*, a common species in grain storage, *C. angustus* has development potential but has been less reported. The finite rate of increase (*λ*) of *C. angustus* was 1.028 at 28 °C in air, which was lower than that of *T. castaneum* (approximately 1.03) [61]. *C. angustus* laid 29 eggs per female at 28 °C in ambient air, which was similar to the 28 eggs per female reported for *T. castaneum* [62]. The adult body size of *C. angustus* is larger, reaching approximately 6 mm in length, whereas *T. castaneum* adults are typically 3–4 mm in length [63]. The values of *R*_0_, *r*, and *λ* decreased with increasing carbon dioxide at both tested temperatures, and the lowest values of *r* and *λ* were detected at 4% carbon dioxide. The population growth potential of *C. angustus* was suppressed at sublethal carbon dioxide; the values of *r* and *λ* decreased, indicating a slower expected population growth rate, whereas the value of *R*_0_ decreased, indicating fewer offspring produced per generation. The developmental duration can be shortened in the tested carbon dioxide; the reproductive ability and population growth of *C. angustus* can be inhibited in the meantime. Although surviving individuals developed quickly, the decreases in survival and fecundity observed here reduced overall population performance. From a practical perspective, the reduced life table parameters (*R*_0_, *r*, *λ*, and *T*) imply a lower risk under elevated low carbon dioxide and support referring to the exposure time of fumigation or controlled atmosphere in practice.

The effectiveness of phosphine fumigation for several highly resistant stored product pests, such as *Sitophilus oryzae* (L.), *Tribolium castaneum* (Herbst), and *Rhyzopertha dominica* (F.) was significantly enhanced by approximately 5% carbon dioxide [4]. Exposure to 4% carbon dioxide under hypoxic conditions led to more pronounced water loss and energy reserve alterations in *P. interpunctella* larvae, suggesting an escalation in physiological stress [64]. The results of decreased survival and developmental duration of *C. angustus* at the tested concentrations of carbon dioxide may have some influence on the insect killing during fumigation and controlled atmosphere.

Temperature increasing by some degree may accelerate insect development and movement, and affect population dynamics by influencing fecundity, survival, generation duration, population size, and geographic range [41]. Undoubtedly, temperature has profound effects on biological traits at all levels of insect organization [65], but at different levels [48]. The two temperatures tested here represented a suitable developmental (28 °C) and low temperature grain storage (23 °C) that was practiced more and more in the modern grain storage industry. Our results identified the sharp difference between 23 °C and 28 °C in the survival and development of this species in the control and treatments. The shortening of developmental duration of *C. angustus* under sublethal carbon dioxide may be associated with prolonged spiracle opening, which could lead to increased water and energy loss and ultimately higher mortality [66]. Through increasing the respiration rate of insects, improving the penetration rate of fumigant and reducing insect tolerance to fumigant, exposure times of controlled atmosphere or fumigation should be shortened, accompanied by carbon dioxide levels similar to the tested concentration [29].

In conclusion, *C. angustus* can finish its whole generation from egg to adult and with a survival rate decreasing to 69% at 23 °C and 74% at 28 °C in carbon dioxide at 0.25–4%. The survival rate, development duration of larvae and total generation, oviposition rate, sex ratio and some life table parameters can obviously be decreased at 0.25% or 0.5% to 4% carbon dioxide. In which the significant changes can be affected by temperature differences. With the survival rate decreasing, *C. angustus* developed more quickly in an environment of 0.25% or 0.5% to 4% of carbon dioxide concentration. Notably, although the development of surviving individuals tended to be quicker under increasing carbon dioxide, the concurrent reductions in survival and reproduction indicate an overall suppression of population growth potential. These results remind us that a biological knowledge of such carbon dioxide conditions should be considered, which may inform exposure time for fumigation or controlled atmosphere strategies.

## Figures and Tables

**Table 1:** Developmental duration and longevity (days) of different life stages of *C. angustus* under the tested carbon dioxide concentrations at 23 °C.

Life Stage	Control	0.25%	0.5%	1%	2%	4%
Egg	4.12 ± 0.07 a	4.06 ± 0.48 a	3.89 ± 0.14 a	3.79 ± 0.15 a	3.65 ± 0.08 a	3.51 ± 0.06 a
L1	3.82 ± 0.18 a	3.73 ± 0.24 a	3.63 ± 0.22 a	3.52 ± 0.23 a	3.34 ± 0.34 a	3.17 ± 0.15 b
L2	5.23 ± 0.13 a	5.07 ± 0.14 a	4.92 ± 0.15 a	4.77 ± 0.11 a	4.51 ± 0.11 b	4.37 ± 0.10 b
L3	6.62 ± 0.21 a	6.44 ± 0.25 a	6.26 ± 0.19 a	6.14 ± 0.15 a	5.93 ± 0.23 a	5.55 ± 0.18 b
L4	6.58 ± 0.05 a	6.38 ± 0.43 a	6.18 ± 0.61 a	5.98 ± 0.03 a	5.63 ± 0.35 a	5.38 ± 0.46 b
L5	7.07 ± 0.72 a	6.86 ± 0.21 a	6.65 ± 0.22 a	6.45 ± 0.74 a	6.39 ± 0.88 a	5.87 ± 0.36 b
L6	6.93 ± 0.14 a	6.72 ± 0.16 a	6.54 ± 0.31 a	6.13 ± 0.66 a	5.95 ± 0.29 a	4.74 ± 0.34 b
L7	6.67 ± 0.77 a	6.46 ± 0.54 a	6.25 ± 0.12 a	6.05 ± 0.83 a	5.65 ± 0.98 a	5.44 ± 0.15 b
L8	8.34 ± 0.34 a	8.08 ± 0.93 a	7.82 ± 0.52 a	7.56 ± 0.46 a	7.75 ± 0.34 a	6.89 ± 0.29 b
L9	6.86 ± 0.15 a	6.65 ± 0.22 a	6.44 ± 0.83 a	6.24 ± 0.71 a	6.02 ± 0.47 a	5.62 ± 0.76 b
L10	7.24 ± 0.21 a	7.02 ± 0.18 a	6.88 ± 0.24 a	6.68 ± 0.19 a	6.46 ± 0.51 a	6.05 ± 0.31 b
L11	7.31 ± 0.26 a	7.09 ± 0.29 a	6.87 ± 0.94 a	6.47 ± 0.41 a	6.22 ± 0.62 a	6.01 ± 0.61 b
L12	6.99 ± 0.15 a	6.78 ± 0.04 a	6.52 ± 0.49 a	6.35 ± 0.22 a	6.02 ± 0.97 a	5.01 ± 0.54 b
Total larval stage	79.68 ± 1.15 a	78.62 ± 1.01 a	77.56 ± 1.05 a	75.22 ± 1.23 ab	71.45 ± 1.08 b	69.39 ± 1.48 c
Pupa	6.97 ± 0.49 a	6.91 ± 0.81 a	6.85 ± 0.59 a	6.79 ± 0.55 a	6.68 ± 0.16 b	6.67 ± 0.12 b
Generation duration	91.74 ± 2.11 a	90.05 ± 2.05 a	89.43 ± 2.58 a	89.12 ± 1.95 a	87.49 ± 1.87 b	87.18 ± 1.82 b
Male longevity	96.59 ± 0.32 a	95.06 ± 0.28 a	94.34 ± 0.21 a	92.13 ± 0.34 a	89.72 ± 0.32 b	87.44 ± 0.41 b
Female longevity	95.07 ± 0.29 a	93.84 ± 0.31 a	92.92 ± 0.27 a	91.68 ± 0.23 a	87.51 ± 0.36 b	85.26 ± 0.34 b

Note: L1–L12 represent the 1st larval instars to the 12th larval instars. Total larval stage included total larval period (L1–L12). Generation duration was the time from egg to adult emergence. Male and female longevity refer to adult lifespan (days) after adult emergence. All data are presented as means ± SE (100,000 bootstrap resampling). Mean values followed by different letters within a row are significantly different according to the paired-bootstrap test (*p* < 0.05).

**Table 2:** Developmental duration and longevity (days) of life stages of *C. angustus* under the tested concentrations of carbon dioxide at 28 °C.

Life Stage	Control	0.25%	0.5%	1%	2%	4%
Egg	3.15 ± 0.74 a	2.98 ± 0.21 a	2.87 ± 0.17 a	2.72 ± 0.34 a	2.51 ± 0.23 a	2.46 ± 0.65 a
L1	3.18 ± 0.05 a	3.14 ± 0.09 a	3.03 ± 0.61 a	2.76 ± 0.14 a	2.61 ± 0.07 a	2.56 ± 0.64 a
L2	3.32 ± 0.31 a	3.26 ± 0.14 a	2.99 ± 0.12 ab	2.94 ± 0.35 a	2.73 ± 0.04 b	2.62 ± 0.19 b
L3	4.89 ± 0.46 a	4.68 ± 0.57 a	4.49 ± 0.38 a	4.29 ± 0.29 a	3.88 ± 0.92 b	3.82 ± 0.65 b
L4	5.63 ± 0.22 a	5.27 ± 0.79 a	5.20 ± 0.48 a	5.07 ± 0.03 a	4.76 ± 0.31 b	4.37 ± 0.84 b
L5	5.16 ± 0.98 a	5.15 ± 0.19 a	4.93 ± 0.46 a	4.59 ± 0.92 a	4.48 ± 0.25 a	4.19 ± 0.87 b
L6	5.68 ± 0.15 a	5.53 ± 0.02 a	5.41 ± 0.72 a	5.19 ± 0.28 a	4.95 ± 0.18 a	4.64 ± 0.64 b
L7	5.25 ± 0.18 a	5.24 ± 0.58 a	5.01 ± 0.45 a	4.88 ± 0.17 a	4.56 ± 0.39 b	4.51 ± 0.24 b
L8	5.42 ± 0.19 a	5.23 ± 0.37 a	5.18 ± 0.45 a	5.02 ± 0.47 a	4.79 ± 0.14 a	4.48 ± 0.61 b
L9	5.68 ± 0.74 a	5.31 ± 0.28 a	5.26 ± 0.73 a	5.09 ± 0.59 a	4.68 ± 0.48 b	4.46 ± 0.12 b
L10	6.45 ± 0.88 a	6.35 ± 0.36 a	6.18 ± 0.46 a	5.97 ± 0.98 a	5.62 ± 0.65 b	5.33 ± 0.07 b
L11	6.59 ± 0.33 a	6.48 ± 0.87 a	6.31 ± 0.89 a	6.11 ± 0.28 a	5.72 ± 0.14 b	5.44 ± 0.63 b
L12	6.90 ± 0.09 a	6.79 ± 0.14 a	6.61 ± 0.42 a	6.40 ± 0.75 a	6.21 ± 0.69 a	5.71 ± 0.48 b
Total larval stage	69.10 ± 1.03 a	68.89 ± 1.34 a	65.81 ± 1.29 a	61.32 ± 1.27 b	60.13 ± 1.26 b	56.41 ± 1.24 c
Pupa	5.62 ± 0.18 a	5.53 ± 0.62 a	5.45 ± 0.21 a	5.19 ± 0.51 a	5.03 ± 0.37 a	4.85 ± 0.63 a
Generation duration	85.54 ± 1.27 a	83.91 ± 1.23 a	80.77 ±1.14 ab	80.30 ± 1.13 b	79.24 ± 1.01 b	79.09 ± 2.01 b
Male longevity	108.08 ± 0.26 a	107.86 ± 0.24 a	105.64 ± 0.25 a	103.43 ± 0.11 a	101.22 ± 0.24 b	98.01 ± 0.19 b
Female longevity	107.72 ± 0.22 a	106.61 ± 0.37 a	104.58 ± 0.18 a	102.39 ± 0.32 a	99.28 ± 0.37 b	97.17 ± 0.15 b

Note: L1–L12 represent the 1st larval instars to the 12th larval instars. Total larval stage represents the total larval period (L1–L12). Generation duration was the time from egg to adult emergence. Male and female longevity refer to adult lifespan (days) after adult emergence. All data are presented as means ± SE (100,000 bootstrap resampling). Mean values followed by different letters within a row are significantly different according to the paired-bootstrap test (*p* < 0.05).

**Table 3:** Survival rates (%) of different life stages of *C. angustus* in concentrations of carbon dioxide at 23 °C.

Life Stage	Control	0.25%	0.5%	1%	2%	4%
Egg	94.72 ± 1.02 a	94.10 ± 0.96 a	93.33 ± 0.88 ab	92.67 ± 0.92 ab	91.85 ± 0.86 b	91.12 ± 0.94 b
L1	91.38 ± 0.65 a	90.62 ± 0.72 a	88.67 ± 0.69 b	86.67 ± 0.81 bc	85.01 ± 0.85 c	84.65 ± 0.85 c
L2	89.36 ± 1.12 a	88.09 ± 1.19 a	85.05 ± 0.51 b	84.67 ± 0.54 b	84.82 ± 0.61 b	83.05 ± 0.76 b
L3	89.21 ± 0.39 a	87.16 ± 0.51 ab	84.15 ± 1.26 bc	83.12 ± 1.35 c	82.95 ± 1.47 c	82.33 ± 1.56 c
L4	88.94 ± 0.25 a	86.40 ± 0.46 b	84.21 ± 0.50 bc	82.67 ± 0.55 c	82.02 ± 0.47 c	81.26 ± 0.18 c
L5	88.77 ± 0.19 a	85.67 ± 0.36 b	83.30 ± 0.54 bc	81.88 ± 0.60 c	81.09 ± 0.63 c	80.82 ± 0.66 c
L6	88.58 ± 0.21 a	85.02 ± 0.41 b	84.62 ± 0.30 b	80.83 ± 0.63 c	80.49 ± 0.38 c	79.61 ± 0.69 c
L7	88.04 ± 0.15 a	84.85 ± 0.24 b	84.43 ± 0.56 b	80.56 ± 0.35 c	80.33 ± 0.65 c	78.18 ± 0.43 d
L8	87.39 ± 0.70 a	84.33 ± 0.49 b	83.95 ± 0.76 b	79.33 ± 0.56 c	78.41 ± 0.29 c	77.67 ± 0.43 c
L9	86.52 ± 0.22 a	83.62 ± 0.53 b	82.43 ± 0.63 b	78.92 ± 0.58 c	77.33 ± 0.49 c	76.82 ± 1.55 c
L10	86.26 ± 0.65 a	82.30 ± 0.74 b	81.95 ± 0.28 b	77.87 ± 1.34 c	76.74 ± 1.46 c	75.10 ± 1.08 c
L11	85.73 ± 0.11 a	81.63 ± 0.56 b	79.18 ± 0.63 c	76.10 ± 0.67 d	75.47 ± 0.70 d	74.24 ± 0.55 d
L12	85.42 ± 0.16 a	80.89 ± 0.58 b	78.41 ± 0.62 c	75.64 ± 0.70 d	74.78 ± 0.76 d	73.93 ± 0.82 d
Total larval stage	84.67 ± 0.78 a	79.84 ± 0.65 b	77.48 ± 1.28 c	75.39 ± 0.64 c	74.56 ± 0.71 c	73.25 ± 0.78 c
Pupa	79.42 ± 0.13 a	77.32 ± 0.66 ab	75.25 ± 0.35 bc	74.10 ± 0.75 c	73.58 ± 0.76 c	73.16 ± 0.25 c
Generation duration	77.63 ± 1.15 a	76.92 ± 1.21 a	73.48 ± 1.28 b	71.87 ± 1.34 bc	70.74 ± 1.46 c	69.82 ± 1.55 c

Note: L1–L12 represent the 1st larval instars to the 12th larval instars. Total larval stage represents the total larval period (L1–L12). Generation duration survival rates were the proportion of individuals surviving from egg to adult emergence. All data are presented as means ± SE (100,000 bootstrap resampling). Mean values followed by different letters within a row are significantly different according to the paired-bootstrap test (*p* < 0.05).

**Table 4:** Survival rates (%) of different life stages of *C. angustus* in concentrations of carbon dioxide at 28 °C.

Life Stage	Control	0.25%	0.5%	1%	2%	4%
Egg	96.55 ± 0.81 a	96.87 ± 0.79 a	96.21 ± 0.84 a	95.52 ± 0.92 a	95.27 ± 0.58 a	94.16 ± 1.02 b
L1	94.68 ± 0.12 a	93.32 ± 0.18 a	92.94 ± 0.21 b	92.61 ± 0.52 b	92.23 ± 0.27 b	90.87 ± 0.31 c
L2	92.21 ± 0.90 a	92.94 ± 0.83 a	90.68 ± 0.46 b	90.25 ± 0.73 b	89.91 ± 0.37 b	87.58 ± 0.85 c
L3	92.07 ± 0.48 a	90.74 ± 0.61 b	89.41 ± 0.52 b	88.04 ± 0.58 c	87.74 ± 0.29 c	84.42 ± 0.63 d
L4	91.98 ± 0.43 a	89.61 ± 0.51 b	88.62 ± 0.26 b	86.21 ± 0.71 c	85.84 ± 0.45 c	82.65 ± 0.71 d
L5	91.61 ± 0.17 a	89.02 ± 0.23 b	87.38 ± 0.52 c	86.15 ± 0.84 c	84.89 ± 0.31 d	82.64 ± 0.25 e
L6	91.33 ± 0.38 a	88.87 ± 0.12 b	87.91 ± 0.03 b	85.84 ± 0.23 c	84.68 ± 0.57 c	81.51 ± 0.11 d
L7	90.67 ± 0.62 a	88.72 ± 0.64 a	86.31 ± 0.19 b	84.43 ± 0.71 c	82.15 ± 0.98 c	80.92 ± 0.19 d
L8	90.51 ± 0.29 a	88.56 ± 0.11 a	86.28 ± 0.21 b	84.01 ± 0.51 c	82.28 ± 0.18 c	78.05 ± 0.25 d
L9	89.87 ± 0.08 a	88.27 ± 0.13 a	85.33 ± 0.18 b	83.36 ± 0.21 c	81.14 ± 0.27 c	78.92 ± 0.31 d
L10	89.79 ± 0.16 a	87.54 ± 0.18 b	85.16 ± 0.20 c	82.18 ± 0.63 c	80.97 ± 0.31 d	77.76 ± 0.35 e
L11	89.33 ± 0.66 a	86.55 ± 0.71 b	83.21 ± 0.25 c	80.85 ± 0.46 d	79.48 ± 0.79 d	77.23 ± 1.03 d
L12	88.67 ± 0.19 a	87.33 ± 0.15 a	82.05 ± 0.17 b	80.81 ± 0.19 c	78.54 ± 0.39 c	77.12 ± 0.22 d
Total larval stage	88.12 ± 1.05 a	84.64 ± 1.12 b	81.24 ± 1.18 c	80.06 ± 1.22 c	77.02 ± 1.27 d	75.94 ± 1.35 e
Pupa	83.11 ± 0.59 a	84.02 ± 0.26 a	80.88 ± 0.47 b	79.43 ± 0.68 b	76.51 ± 0.69 c	74.68 ± 0.35 d
Generation duration	81.05 ± 1.41 a	80.47 ± 1.32 a	79.51 ± 1.42 a	78.28 ± 1.51 b	75.06 ± 1.64 c	74.05 ± 1.72 c

Note: L1–L12 represent the 1st larval instars to the 12th larval instars. Total larval stage represents the total larval period (L1–L12). Generation duration survival rates were the proportion of individuals surviving from egg to adult emergence. All data are presented as means ± SE (100,000 bootstrap resampling). Mean values followed by different letters within a row are significantly different according to the paired-bootstrap test (*p* < 0.05).

**Table 5:** Sex ratio of *C. angustus* at different carbon dioxide concentrations.

CO_2_ (%)	23 °C	28 °C
Control	1.19 ± 0.03 aA	1.21 ± 0.07 aA
0.25	1.14 ± 0.03 bA	1.17 ± 0.04 aA
0.5	1.13 ± 0.03 bA	1.15 ± 0.04 aA
1	1.07 ± 0.04 cA	1.12 ± 0.06 abA
2	0.99 ± 0.03 dA	1.08 ± 0.09 bA
4	0.87 ± 0.02 eA	0.93 ± 0.02 cB

Note: All data are presented as means ± SE (100,000 bootstrap resampling). Mean values followed by different lowercase letters within a column are significantly different according to paired-bootstrap test (*p* < 0.05). Mean values followed by different uppercase letters within a row are significantly different between temperatures within the same carbon dioxide concentration, based on an independent samples *t*-test (*p* < 0.05).

**Table 6:** Oviposition and life table parameters of *C. angustus* under different carbon dioxide concentrations at 23 °C.

Parameters	Control	0.25%	0.5%	1%	2%	4%
Pre-oviposition (days)	8.08 ± 0.52 a	8.27 ± 0.17 a	8.45 ± 0.31 ab	8.62 ± 0.17 b	8.85 ± 0.06 b	8.95 ± 0.43 c
Oviposition duration (days)	26.36 ± 0.59 a	25.92 ± 0.82 a	25.83 ± 0.75 a	25.36 ± 0.48 a	24.93 ± 0.65 b	24.58 ± 0.78 b
Eggs laid per female	28.48 ± 0.72 a	26.06 ± 0.59 b	24.84 ± 0.83 bc	22.63 ± 0.41 c	21.52 ± 0.67 d	19.02 ± 0.76 e
*R*_0_	12.44 ± 0.18 a	10.66 ± 0.15 b	9.67 ± 0.22 c	8.39 ± 0.13 d	7.45 ± 0.19 e	6.16 ± 0.16 f
*r*	0.0253 ± 0.0012 a	0.0241 ± 0.0011 a	0.0231 ± 0.0014 b	0.0216 ± 0.0015 c	0.0208 ± 0.0024 cd	0.0194 ± 0.0015 d
*λ*	1.0256 ± 0.0023 a	1.0244 ± 0.0027 a	1.0234 ± 0.0028 b	1.0218 ± 0.0024 c	1.0210 ± 0.0031 cd	1.0196 ± 0.0026 d
*T*	99.82 ± 1.84 a	98.32 ± 2.01 ab	97.88 ± 1.95 b	97.74 ± 2.12 b	96.34 ± 1.76 c	96.11 ± 1.88 c

Note: *R*_0_: net reproductive rate, *r*: intrinsic rate of increase, *λ*: finite rate of increase, *T*: mean generation time. All data are presented as means ± SE (100,000 bootstrap resampling). Mean values followed by different letters within a row are significantly different according to paired-bootstrap test (*p* < 0.05).

**Table 7:** Oviposition and life table parameters of *C. angustus* under different carbon dioxide concentrations at 28 °C.

Parameters	Control	0.25%	0.5%	1%	2%	4%
Pre-oviposition (days)	6.98 ± 0.13 a	7.01 ± 0.08 a	7.08 ± 0.14 a	7.15 ± 0.20 a	7.59 ± 0.15 b	7.91 ± 0.72 b
Oviposition duration (days)	28.88 ± 0.32 a	28.15 ± 0.48 a	28.04 ± 0.55 a	27.29 ± 0.56 a	26.82 ± 0.62 b	26.36 ± 0.27 b
Eggs laid per female	29.72 ± 0.81 a	26.39 ± 0.62 b	25.98 ± 0.79 b	23.55 ± 0.36 c	22.63 ± 0.68 c	20.52 ± 0.85 d
*R*_0_	13.13 ± 0.25 a	11.45 ± 0.21 b	10.87 ± 0.19 b	9.74 ± 0.22 c	8.53 ± 0.17 d	7.34 ± 0.20 e
*r*	0.0278 ± 0.0015 a	0.0268 ± 0.0017 a	0.0271 ± 0.0013 a	0.0260 ± 0.0019 ab	0.0246 ± 0.0018 b	0.0232 ± 0.0013 c
*λ*	1.0282 ± 0.0021 a	1.0272 ± 0.0026 a	1.0275 ± 0.0028 a	1.0263 ± 0.0034 ab	1.0249 ± 0.0036 b	1.0235 ± 0.0027 c
*T*	92.52 ± 1.40 a	90.92 ± 1.31 a	87.85 ± 1.28 b	87.45 ± 1.33 b	86.83 ± 1.16 b	86.91 ± 1.45 b

Note: *R*_0_: net reproductive rate, *r*: intrinsic rate of increase, *λ*: finite rate of increase, *T*: mean generation time. All data are presented as means ± SE (100,000 bootstrap resampling). Mean values followed by different letters within a row are significantly different according to paired-bootstrap test (*p* < 0.05).

## Data Availability

The original contributions presented in the study are included in the article, further inquiries can be directed to the corresponding author.
